# Health-related quality of life among Jewish older persons in Mexico and its determinants

**DOI:** 10.1186/s12955-020-01401-4

**Published:** 2020-05-25

**Authors:** Mariana López-Ortega, Mina Konigsberg

**Affiliations:** 1 Research Department National Institute of Geriatrics National Institutes of Health, Mexico City, Mexico; 2 Health Sciences Department, Autonomous Metropolitan University, Iztapalapa, Mexico City, Mexico

**Keywords:** Older persons, Jewish community, Health-related quality of life, Mexico

## Abstract

**Purpose:**

Aging research in Mexico has significantly increased in the past decades, however, little is known on health related quality of life (HRQoL) of older adults. The aim of this study was to expand this field by examining HRQL in a representative sample of Jewish older adults in Mexico, and to investigate its association with different factors.

**Methods:**

This was a cross-sectional survey of a random sample of community dwelling Jewish men and women aged 60 years and older. HRQoL was measured using the Short Form Health Survey (SF-36). Bivariate analysis was performed to estimate the association of scores of HRQoL and different characteristics of the study sample and multiple linear regression models were estimated using ordinary least squares (OLS), to explore determinant factors associated to HRQoL in this sample, for the eight domains of the SF-36 sub-scales separately.

**Results:**

Two hundred ninety-five older persons were interviewed. Mean age was 72.7 years (SD 7.9), men made up 57% of the sample, 67% were married and 52% reported living with another person, mostly the spouse. Higher HRQoL was associated with higher educational attainment, being married, and having higher social support, while lower HRQoL was associated with being widowed, in worse financial situation, having chronic diseases and being in the oldest age groups.

**Conclusions:**

Findings show that gender, socioeconomic level, educational attainment, marital status as well as social support & community participation are relevant factors influencing HRQoL in our study sample. With respect to the SF-36 subscales, HRQoL of Jewish older adults in Mexico present higher scores than that of adults and older adults previously found in other studies in Mexico. Further studies comparing other characteristics among them could help bring further understanding of these differentiated ageing processes.

## Background

Mexico is going through a demographic transition that is leading to a rapidly ageing population. This process has occurred in parallel to fundamental social and economic changes. While different theories on ageing have been developed, it is clear that while longevity has increased worldwide, for an important number of older persons, especially in low-and middle-income countries like Mexico, this process is experienced alongside disabilities, economic hardship, increasing dependence on others to perform daily activities, and limited access to formal health and personal care services [1]. In this context, it becomes necessary to, not only characterize health status and socioeconomic conditions, but to investigate how people experience old age and their ageing process. It is not about adding years to life but to insure that years gained can be lived with quality. Ideally, then, quality of life (QoL) through the life course and as one reaches old age, should be a continuous subject of study and its results used as input in the generation of specific health and ageing strategies.

QoL is a broad concept that includes general and individual characteristics and has diverse definitions and valuation methods. It is a multidimensional concept that generally includes objective and subjective domains, is related to the individual’s perception of his or her position in life within the context of her or his culture and value system and with relation to his or her goals, expectations and principles [2–4]. There is no universal definition of QoL, and given its multidimensional nature, its valuation or measurement has to inquire a wide range of domains of personal life such as physical and psychosocial wellbeing, functional status and disability, among others [5].

As health research has increased, interest in shifting the focus in public health towards health promotion and QoL has advanced quickly, increasingly focusing on understanding how to improve not only health outcomes, but also health-related quality of life (HRQoL) [6]. Several conceptual models of HRQoL have been developed in the last decades, incorporating physical and mental health, individual and environmental factors as main determinants of HRQoL. In this study, we followed one of the most widely used conceptual models developed by Wilson and Clearly [7], and the revision of this model by Ferrans and colleagues [8], considering them to be comprehensive in terms of the factors and links most relevant to HRQoL. Briefly, the model is composed by five core domains including biological and physiological factors, symptoms, functional capacity, general health perceptions, and perceived QOL. In addition, characteristics of the social environment such as social support and marital status are also considered to influence the main domains and are included in the model [7]. The revision by Ferrans and colleagues maintains the five core domains and further developed the definitions for individual and environmental characteristics [8].

In addition, different measures have been developed to evaluate general and specific features of HRQoL considering differences in age, population subgroups, etc. It is considered that HRQoL can be used to evaluate multidimensional population health outcomes better than general QoL, supplementing traditional measures of mortality and morbidity, and providing a broad summary measurement of perceived health. HRQoL constructs include measures of physical health, mental health, and social functioning, and a vast number of studies have empirically tested different instruments while others have focused on the translation and validation of these in different languages [9–11]. Previous studies have identified socio-economic and health related features such as education, number of chronic diseases, self-perceived symptoms of depression and difficulties with performing daily activities [12–18] as determinants of general QoL and HRQoL.

One of the most widely used instruments to measure HRQoL is the Medical Outcomes Study (MOS) Short-Form-36 Health Survey, SF-36 [19]. This instrument addresses health concepts that are relevant to patients from the patient’s perspective. The predictive validity of the SF-36 has been documented by the International Quality of Life Assessment Project (IQOLA) who first translated, validated and adapted the SF-36 in seven European countries, followed by its application in more than 40 countries [19–21]. This instrument allows the measurement of different health dimensions, can assess the impact of illness and treatments between subjects, and has been an appropriate instrument to assess HRQoL in adults and a good predictor of mortality [19, 22]. The survey was constructed for self-administration by persons 14 years of age and older and for administration by a trained interviewer in person or by telephone [22].

In Mexico, significant progress has been made in the past 20 years to characterise health conditions, disability, morbidity and mortality of Mexican older persons; however, much less research has been conducted examining their HRQoL. To date, there are two nationally representative surveys of older persons which have greatly influenced progress in aging research, one longitudinal -the Mexican Health and Aging Study (MHAS), and a cross-sectional survey -the National Health and Nutrition Survey (ENANUT) 2012. However, these surveys do not allow for the detailed study of subgroups of the population, such as minorities or indigenous groups. Within these, the Jewish Community in Mexico (JCM) is an interesting case study given that it is estimated that adults 60 years and older represent 22% percent of total population in that community, compared to 10.5% nationally where this group is expected to reach the same 20% by the year 2030 [23]. In addition, it allows for comparison with national level data and with Jewish older persons in other countries. Therefore it is interesting to understand how this minority group has managed to have such a high percentage of older adults and evaluate their HRQoL.

The aim of the present study was to provide a profile of the SF-36 test in older persons of the Jewish community in Mexico and to analyse the impact of demographic, socioeconomic and health conditions on HRQOL in this population group. In the study, the term Jewish refers to an ethnoreligious and ethno-cultural group of individuals that belong to the Jewish Community in Mexico (JCM) and that come from two ethnic ascendants: Eastern Europe (Ashkenazim Jews, 47%), and Arabic Countries and Turkey (Sephardim Jews 53%); and are registered with the Jewish Community Central Committee (CC) which gathers diverse information on the community and generates a global registry.

While there are an important number of studies on the Jewish immigration process to Mexico and their assimilation [24–28], this study is considered of high relevance given the limited detailed information available from ethnic minority groups in general and specifically of the Jewish community in Mexico, giving us the opportunity to advance our knowledge on their demographic characteristics, their socioeconomic conditions and their health status, including their HRQoL.

## Methods

### Study design and data collection

Data were obtained from the *Study of Health and Well-being of Older Persons of the Mexican Jewish Community* (Estudio sobre la salud y bienestar de los adultos mayores de la comunidad Judía de México, ESABIAM-CJM). This was a cross-sectional survey of a random sample of community-dwelling Jewish adults 60 years and older living in Mexico City. In order to estimate the representative sample we approached the Mexican Jewish Central Committee (MJCC), the stewardship institution that centralises all institutions within the Jewish community in Mexico, conducts an internal census of its community members, and keeps a registry at the individual level.

Information provided by the MJCC stated 43,000 Jews living in Mexico City and its metropolitan area (MCMA) in the year 2014, of which 22% (*n* = 9460) were adults 60 years and older. While some families are registered in few other cities in the country, according to internal studies by the MJCC, it is estimated that 98% of total Jewish population lives within MCMA. Given the interest of studying this population independently, simple random sampling methods were used to allow each older adult registered with the MJCC the same chance and likelihood of being selected. In order to obtain the needed sample, we used the standard formula for population sample size calculation determining a 95% confidence level, a 50% standard deviation, and a margin error of 5%. Applying the formula, the estimated total number of older adults needed to obtain a representative population sample was 370 individuals 60 years and older.

In order to select our sample, the MJCC provided a blinded list of all individuals 60 years and older registered in the community and each individual (blinded record) was given a number. From the blinded list, first, 407 numbers were randomly selected using a computer program to identify the 370 individuals needed in order to obtain a representative sample, plus a 10% to account for non-response. Secondly, 20 additional numbers were selected in order to conduct a pilot of the questionnaire.

After the standardization of the questionnaire was performed, and once the blinded numbers were selected, they were provided to the MJCC who then sent personalised invitation letters to all selected respondents followed by telephone appointments. Once confirmed, all participants were interviewed at their home, those not available for the interview at the moment of the appointment were asked for further appointments until the interview or a full refusal was obtained. Data was collected from January to September 2016 through a standardized questionnaire by previously trained personnel, and supervised by the principal investigators of the study.

The Ethics Committee at the National Institute of Geriatrics reviewed and approved the study (SIRES-DI-JEDDS-002/14). All participants and a witness individually signed a consent letter that was previously read aloud by the interviewer. In addition, each participant received printed information with contact details of the principal investigators and the Ethics Committee at the Institute, in case they had any questions about the project or their participation.

### Measurements

A survey questionnaire was developed to capture all information. The survey included five sections according to the topics of interest: socioeconomic and demographic characteristics, family support and social networks, health insurance and service utilisation, self-report of time spent in different daily activities, and HRQoL.

### Outcome measure

Health-related quality of life (HRQoL) was measured using the MOS 36-item Short Form Health Survey SF-36 [22]. The instrument includes 36 items to assess functional health and well-being from the perspective of the patient (self-reported). It includes one multi-item scale that assesses eight health concepts: PF-Physical functioning; (10 items), RP-Role limitations due to physical health problems (four items), RE-Role limitations due to emotional problems (three items), VT-Vitality, energy and fatigue (four items), MH-Mental health including psychological distress and emotional well-being (five items), SF-Social functioning (two items), BP-Bodily Pain (two items), and GH-General health perceptions (five items) [22]. This test has been previously validated in Mexico [29] and normative data for Mexican adults has been previously reported [30].

Scoring was performed using the normal additive approach that produces scores that range from 0 to 100 for the eight scales, with higher scores indicating better HRQoL [31]. Instrument reliability analysis was performed by evaluating the internal consistency of the test, using Cronbach’s alpha as well as item-test correlations. In addition, Pearson correlations among all of the items on the scale (inter-item) were performed. Consistent with previous research, the SF-36 presented high reliability in this study. In each of the eight dimensions, the SF-36 had an alpha greater than 0.80, a total alpha of 0.86 and item-total correlation of 0.62 or higher for each item of the scale (detailed results available upon request).

### Covariates

Socioeconomic and demographic data included sex, age, marital status (single including divorced or separated, married/partnered, widowed), educational attainment, self-reported financial situation and main occupation. From recorded age, an age group variable was generated including three groups: 60–69 years, 70–79 and 80 years and above. Educational attainment is presented as total number of years of formal schooling. While objective measures of financial status could enrich the analysis, subjective socio-economic and financial status has been shown to be a better predictor of health status and decline in health status over time [32]. Thus, in order to assess economic status, self-reported financial situation was assessed with the question: Would you say that you financial situation is..., with the following response options: Excellent, very good, good, fair, bad, and very bad. For the analyses, these were categorised into four groups: very good, good, fair, and poor.

Main occupation included three categories: working, retired/pensioner, and domestic/household work. Social support was measured with two variables. First, one variable for living arrangements captures all persons living in the household in addition the respondent. The variable also includes zero for those who declared no additional household members, that is, reported living alone. The second variable, personal contacts with family or friends in the past week, is a categorical variable accounting for a self-report of the total number of personal contacts that each respondent had in the past week: None, 1 to 3, 3 to 5, and More than 5. For the regression analyses, a dummy variable was generated with categories of up to 3 contacts (including no contacts), and more than three contacts in the past week.

Health condition of the respondent was captured in two variables indicating chronic diseases; one continuous variable representing none or up to five of the following: diabetes, hypertension, cancer, stroke and heart attack, and a three categories variable to indicate no chronic diseases, one, and two or more.

### Statistical analysis

Descriptive analyses of sociodemographic and health status of the study sample were performed. Scores for each of the eight dimensions of the SF-36 were coded, summed, and ranked on a scale from worst (zero) to best (100) possible HRQoL status, and estimates of means, standard error and confidence intervals calculated. Bivariate analysis was performed using the Student’s t-test and ANOVA to estimate the association of scores of HRQoL and different characteristics of the study sample. Multiple linear regression models were estimated using ordinary least squares (OLS), to estimate determinant factors of HRQoL for the eight domains of the SF-36 sub-scales separately. To test multiple hypotheses, we applied a Holm-Bonferroni correction [33], and we estimate robust standard errors. All analyses were performed using Stata 14 software [34].

## Results

From the 407 identified individuals in the random sample (370 to obtain representative sample plus 10% to account for non-response), and that were sent personalised invitation letters, 28 individuals could not be contacted because they had moved or were deceased at time of contact, 7 were ill at the moment of contact and could not participate, and 77 refused to participate. For the remaining 295 individuals, complete interviews were obtained, these constituted our working sample for the analyses, corresponding to a total 80% response rate.

Of the total respondents, 43% were women and 21% were not born in Mexico. Mean age was 72.7 years (SD 7.9), most of the interviewees were married or partnered (67.4%), and educational attainment was high compared with older persons at national level with an average of 13 years of formal schooling (SD 4.3 years). In addition, 57.8% of the sample reported they were still working (Table 1) and 46% reported having no chronic illnesses. About two thirds of the sample (65.6%) reported a good or very good financial situation.

**Table 1:** Characteristics of study sample

*N* = 295	% or Mean (SD)
*Gender*	
Men	56.95
Women	43.05
*Age mean (SD)*	72.7 (7.9)
*Age group*	
60–69 years	41.3
70–79 years	36.5
80+ years	22.2
*Marital status*	
Single	12.2
Married/ Partnered	67.4
Widowed	20.4
*Living arrangements*	
Living alone	18.4
Respondent and 1 additional household member	51.9
Respondent and 2 or more household members	29.7
*Place of Birth*	
Mexico	79.0
Another country	21.0
*Years of schooling*	13.1 (4.3)
*Main ocupation*	
Working	57.8
Retired	15.9
Housework	21.5
Other	4.8
*Self-reported financial situation*	
Very good	18.8
Good	46.8
Fair	23.9
Poor	10.6
*Frequency of contact with family or close friends in past week*	
None	3.7
1–3	40.1
3–5	19.0
More than 5	37.1
*Number of chronic diseases*	
0	46.4
1	34.6
2+	19.0

*SD* standard deviation

In this sample, 18% of the respondents reported living alone, while 52% reported living with another person, mostly the spouse or partner. Finally, older persons in the sample appeared to be socially active with approximately 56% reporting having contact with family or close friends 3 or more times in a week.

Scores of HRQoL were lowest in the vitality (68.4), general health (71.9) and mental health (76.5) dimensions, while highest scores were obtained in the dimensions of role-emotional (89.6), role-physical (87.5) and social functioning (87.2). With the exception of general health, women obtained lower scores than men in all domains, with the largest difference observed in the physical functioning scale, with a difference of 7.2 points in mean scores. Differences between men and women in scores of physical functioning scale, vitality and bodily pain were statistically significant (*p* ≤ 0.01) (Table 2).

**Table 2:** Relation between SF-36 domains and selected characteristics of sample subjects

	PF			RP			RE			VT			MH			SF			BP			GH		
	Mean	[95%	CI]	Mean	[95%	CI]	Mean	[95%	CI]	Mean	[95%	CI]	Mean	[95%	CI]	Mean	[95%	CI]	Mean	[95%	CI]	Mean	[95%	CI]
Total (*n* = 294)	77.3	74.3	80.4	87.5	84.0	91.0	89.6	86.3	92.8	68.3	65.9	70.7	76.5	74.3	78.7	87.2	84.7	89.7	81.5	78.8	84.2	71.9	69.8	73.9
*Gender*																								
Male	80.4	76.7	84.1	88.5	84.2	92.8	91.2	87.1	95.3	69.8	66.6	73.0	77.9	75.1	80.7	88.2	84.9	91.4	83.6	80.0	87.3	70.6	67.8	73.4
Female	73.2	68.2	78.3**	86.2	80.3	92.1	87.4	82.0	92.8	66.3	62.7	69.8*	74.6	71.1	78.0	85.9	82.0	89.9	78.8	74.8	82.8**	73.5	70.4	76.6
*Age group*																								
60–69 years	88.6	85.7	91.4	91.5	87.1	95.9	91.2	86.3	96.1	73.5	69.88	77.1	78.0	74.8	81.2	89.3	85.4	93.1	83.3	78.9	87.7	75.9	72.9	79.0
70–79 years	78.7	74.2	83.2	86.3	80.1	92.5	89.6	84.4	94.9	68.8	64.8	72.7	76.6	72.8	80.5	87.4	83.3	91.5	82.9	78.8	87.1	70.4	66.9	73.8
80 years	53.8	45.8	61.8***	81.5	72.4	90.7*	86.2	77.9	94.4	57.8	53.1	62.5***	73.3	68.3	78.3	82.7	76.9	88.5	75.4	69.5	81.2*	66.3	61.7	71.0***
*Marital status*																								
Single	79.0	71.0	87.1	75.0	60.4	89.6	79.0	66.0	92.1	63.3	55.0	71.5	68.2	60.9	75.6	78.9	70.3	87.6	73.1	62.8	83.4	69.3	63.2	75.4
Married/ Partnered	81.3	78.1	84.4	91.7	88.3	95.1	91.6	87.9	95.2	71.01	68.4	73.7	78.6	76.2	81.0	89.71	86.9	92.5	84.3	81.3	87.4	73.0	70.5	75.4
Widowed	62.8	53.8	71.8***	80.8	71.1	90.6**	88.9	81.3	96.4**	62.2	56.3	68.2***	74.1	68.5	79.8***	83.8	77.5	90.0**	77.0	71.3	82.7***	69.6	64.3	74.9
*Living arrangements*																								
Living alone	73.0	64.6	81.3	84.0	74.4	93.5	87.4	78.6	96.3	66.8	61.1	72.4	73.6	67.5	79.8	85.6	79.7	91.5	79.2	72.6	85.7	71.6	66.3	77.0
Respondent and 1 additional household member	77.0	73.0	81.1	87.7	82.8	92.5	87.9	83.1	92.8	67.0	63.6	70.4	77.8	74.9	80.7	86.2	82.5	89.9	81.1	77.4	84.8	71.6	68.8	74.4
Respondent and 2 or more household members	80.1	74.5	85.7	89.1	83.0	95.1	93.5	88.7	98.3	71.3	66.9	75.6	76.3	72.4	80.2	89.9	85.8	94.1	84.2	79.2	89.2	72.5	68.4	76.6
*Place of Birth*																								
Mexico	77.5	74.2	80.9	87.1	83.1	91.0	91.1	87.7	94.5	69.2	66.6	71.8	76.8	74.3	79.3	88.8	86.2	91.5	81.4	78.3	84.4	71.7	69.3	74.1
Another country	76.5	69.1	83.9	89.1	81.5	96.7	83.9	74.8	92.9*	64.8	59.3	70.4	75.2	70.3	80.1	81.0	74.4	87.7**	82.0	76.3	87.7	72.4	68.1	76.6
*Main occupation*																								
Working	84.6	81.6	87.5	88.3	83.9	92.7	91.0	86.9	95.1	69.4	66.2	72.6	76.6	73.7	79.5	88.7	85.6	91.8	82.6	79.0	86.1	72.3	69.5	75.0
Retired	68.9	60.1	77.7	87.0	77.9	96.0	89.1	80.3	98.0	67.3	61.1	73.5	78.1	72.4	83.8	84.5	77.9	91.2	81.3	74.0	88.5	68.3	63.0	73.7
Housework	63.9	55.8	72.0***	85.5	76.5	94.5	84.9	76.5	93.4	66.3	61.2	71.5	74.2	69.2	79.3	86.3	80.2	92.3	77.1	71.2	83.1	71.3	66.6	76.0
*Self-reported financial situation*																								
Very good	86.6	80.9	92.4	95.9	91.8	100.0	95.8	90.5	101.0	76.4	71.7	81.0	84.1	80.5	87.7	93.0	88.9	97.0	87.8	83.2	92.5	76.8	72.4	81.2
Good	79.8	75.6	83.9	88.3	83.1	93.5	92.0	87.6	96.3	70.5	67.3	73.7	79.4	76.8	81.9	89.4	86.2	92.7	83.8	80.2	87.4	73.3	70.5	76.0
Fair	78.2	72.4	84.0	85.7	78.1	93.3	88.1	81.1	95.1	66.1	61.1	71.2	75.0	70.4	79.5	87.5	82.3	92.7	78.7	72.6	84.7	72.4	68.5	76.4
Poor	48.4	37.2	59.5***	72.6	57.5	87.6***	72.0	56.2	87.9***	49.5	41.0	58.0	54.3***	44.8	63.8	66.1***	54.9	77.3***	66.9	55.7	78.0***	56.2	47.4	65.0***
*Social support*																								
Frequency of family of close friends contacts in past week mean (SD)																								
None	77.3	57.9	96.6	88.6	68.3	109.0	78.8	51.8	105.8	56.4	44.7	68.0	70.5	57.8	83.3	79.5	61.8	97.3	88.0	75.6	100.3	75.2	64.1	86.2
1–3	72.4	67.1	77.6	87.5	81.9	93.1	87.0	81.2	92.8	65.4	61.4	69.3	74.7	71.0	78.3	84.1	79.8	88.4	80.9	76.7	85.2	68.5	65.0	71.9
3–5	78.7	72.1	85.3	88.8	81.2	96.5	93.5	87.7	99.2	66.2	61.6	70.7	75.1	71.1	79.0	92.9	89.5	96.2	79.9	73.6	86.2	71.7	67.7	75.7
More than 5	82.0	77.4	86.5**	86.7	80.7	92.7	91.4	86.5	96.4	73.7	69.8	77.6***	79.7	76.0	83.5**	88.4	84.1	92.7**	82.4	77.8	86.9	75.3	71.8	78.8**
*Number of chronic diseases*																								
0	81.2	76.8	85.5	89.9	85.1	94.6	91.9	87.5	96.3	71.3	67.8	74.7	78.0	74.8	81.2	89.2	85.9	92.4	83.6	79.7	87.5	75.1	72.0	78.3
1	77.3	72.2	82.4	85.8	79.4	92.1	87.3	81.0	93.5	66.8	62.5	71.0	75.6	72.1	79.1	86.6	82.2	91.1	80.4	75.8	84.9	72.7	69.7	75.8
2+	68.0	60.5	75.4***	84.8	75.9	93.7	88.1	80.4	95.8	63.9	58.9	68.9**	74.2	68.4	80.0	83.5	76.6	90.3	78.7	72.3	85.1	62.4	57.4	67.3

*: *p* < 0.001; **: *p* < 0.01; ***: *p* < 0.05

*PF* physical functioning, *RP* role limitations due to physical health problems, *RE* role limitations due to emotional problems, *VT* vitality, energy and fatigue, *MH* mental health including psychological distress and emotional well-being, *SF* social functioning, *BP* bodily pain, *GH* general health perception

As age increased, scores in all domains decreased with the highest differences observed between those 80 years and older and the other age groups. Scores were statistically significantly different among age groups for the physical functioning (*p* > 0.05), role physical (*p* < 0.001), bodily pain (*p* < 0.001), and general health dimensions (*p* < 0.05).

Marital status of sample respondents was a statistically significant factor of HRQL with single and widowed respondents reporting lower scores than those who were married (Table 2). In addition, those living alone reported lower scores than those living in households with 2 or more residents; however, these differences were not statistically significant. Statistically significant differences were also found in all dimensions of HRQoL regarding financial situation of the respondent, where individuals with poor financial status reported significantly lower scores (*p* < 0.05). As expected, those with chronic diseases consistently reported lower scores in all dimensions of the SF-36, however, differences were only statistically significant for the physical functioning (*p* < 0.05) and vitality (*p* < 0.01) (Table 2).

For each of the 8 domains, Table 3 shows the independent variables included in each regression. Regression coefficients and 95% Confidence Intervals (CI) are also presented and statistically significant coefficients are indicated in the table.

**Table 3:** Regression results: Determinants of the eight SF-36 domain scores

	PF	RP	RE	VT	MH	SF	BP	GH
	β-coefficient [95% CI]	β-coefficient [95% CI]	β-coefficient [95% CI]	β-coefficient [95% CI]	β-coefficient [95% CI]	β-coefficient [95% CI]	β-coefficient [95% CI]	β-coefficient [95% CI]
**Male**	− 2.7 (−9.6–4.2)	−5.7 (− 15.4–4.0)	2.5 (− 6.7–11.8)	1.7 (− 4.6–7.9)	1.7 (− 4.0–7.5)	− 0.2 (− 6.8–6.5)	0.5 (− 7.1–8.0)	−5.5 (− 11.0–0.1)***
**Age (years)**								
70 to 79	−3.5 (− 9.9–2.9)	− 5.5 (− 14.5–3.5)	3.3 (− 5.2–11.9)	−3.3 (− 9.0–2.5)	−1.1 (− 6.5–4.2)	0.6 (− 5.6–6.8)	1.2 (− 5.8–8.2)	−3.5 (− 8.6–1.6)
80 and above	−20.5 (− 28.3 − − 12.7)* ††	−6.0 (− 17.0–5.1)	4.1 (−6.4 − 14.6)	− 12.7 (− 19.8 − − 5.6)** ††	0.0 (− 6.5–6.6)	−1.8 (− 9.3–5.8)	−4.5 (− 13.1–4.0)	− 3.4 (− 9.7 − 2.9)
**Years of formal education**	0.9 (0.3–1.6)	0.1 (− 0.8–1.1)	1.0 (0.1 − 1.9)***	0.2 (− 0.4–0.8)	0.1 (− 0.5–0.6)	0.1 (− 0.5–0.8)	−0.1 (− 0.9–0.7)	0.3 (− 0.3–0.8)
**Marital status**								
Married/partnered	3.1 (− 5.4–11.7)	21.3 (9.2–33.3)* ††	12.1 (0.7 − 23.6)***	5.6 (− 2.2–13.3)	9.3 (2.2–16.5)**	11.6 (3.4–19.9)** ††	10.1 (0.8–19.5)***	3.8 (−3.0–10.7)
Widowed	−5.2 (− 15.5–5.1)	11.4 (− 3.1–26.0)	15.6 (1.8 − 29.4)***	1.5 (− 7.8–10.9)	7.2 (− 1.4–15.9)	10.2 (0.2–20.1)***	8.7 (− 2.5–20.0)	1.0 (− 7.3–9.3)
**Self-reported financial situation**								
Good	−5.8 (− 13.1–1.5)	−4.9 (− 15.1–5.4)	−0.6 (− 10.3 − 9.2)	− 3.9 (− 10.5–2.7)	− 1.5 (− 7.6–4.6)	−0.3 (− 7.3–6.8)	−2.7 (− 10.7–5.2)	−1.7 (− 7.5–4.2)
Fair	−4.9 (− 13.2–3.4)	−9.2 (− 20.8–2.5)	−2.6 (− 13.7 − 8.5)	− 8.8 (− 16.3 − − 1.3)***	−7.0 (− 13.9 − − 0.1)	− 3.1 (− 11.0–4.9)	− 8.0 (− 17.0–1.1)	−1.7 (− 8.3–5.0)
Poor	−22.0 (− 32.5 − − 11.4)* ††	−21.5 (− 36.3 − − 6.7)** ††	−20.3 (− 34.4 − − 6.2)** ††	−20.5 (− 30.0 − − 11.0)* ††	−26.2 (− 35.0 − − 17.4)* ††	− 25.4 (− 35.5 − − 15.2)* ††	−18.4 (− 29.9 − − 6.9)** ††	−13.1 (− 21.6 − − 4.7)** ††
**Main occupation**								
Retired	−6.6 (− 14.3–1.1)	6.8 (− 4.0–17.7)	−2.8 (− 13.1 − 7.5)	3.8 (− 3.2–10.7)	3.2 (− 3.2–9.7)	−1.2 (− 8.6–6.2)	0.1 (− 8.4–8.4)	−0.3 (− 6.4–5.9)
Housework	−8.6 (− 17.1–0.0)***	−1.1 (− 13.1–11.0)	−4.2 (− 15.6 − 7.2)	5.5 (−2.3–13.2)	0.5 (−6.6–7.6)	−0.3 (− 8.5–8.0)	−4.1 (− 13.4–5.2)	−0.2 (− 7.0–6.7)
**Chronic diseases**								
One	0.1 (−5.8–6.0)	− 1.0 (− 9.3–7.3)	−4.6 (− 12.5 − 3.3)	−2.5 (− 7.9–2.8)	−2.6 (− 7.5–2.3)	−1.5 (− 7.2–4.2)	−3.2 (− 9.6–3.3)	−1.7 (− 6.5–3.0)** ††
Two or more	− 4.3 (− 11.6–3.0)	−3.5 (− 13.8–6.8)	−0.5 (− 10.3 − 9.3)	− 2.9 (− 9.5–3.7)	−1.3 (− 7.4–4.8)	−2.3 (− 9.4–4.7)	−3.9 (− 11.9–4.1)	−10.1 (− 16.0 − − 4.2)
**Number of close contacts last week**	6.7 (1.4–12.0)**	−1.7 (− 9.2–5.7)	5.8 (− 1.2 − 12.9)	5.1 (0.3–9.8)***	1.9 (− 2.5–6.3)	4.7 (− 0.4–9.8)	− 0.4 (− 6.1–5.4)	3.6 (− 0.6–7.9)
**Household members in addition to respondent**	1.1 (−1.5–3.8)	−2.0 (− 5.8–1.7)	0.9 (− 2.7 − 4.5)	0.1 (−2.3–2.5)	−0.3 (− 2.5–1.9)	0.1 (−2.5–2.7)	0.6 (− 2.3–3.5)	1.0 (− 1.1–3.2)
_cons	75.6 (59.3–92.0)	86.6 (63.6–109.5)	63.4 (41.6 − 85.2)	67.3 (52.5–82.0)	72.6 (59.0–86.2)	78.2 (62.5–93.9)	81.5 (63.6–99.4)	72.3 (59.2–85.4)
R-squared	0.40	0.11	0.12	0.21	0.22	0.19	0.11	0.18
Adjusted R-squared	0.37	0.05	0.07	0.17	0.17	0.14	0.05	0.13
F-test (15, 241)	10.9	1.98	2.29	4.4	4.5	3.83	1.96	3.56
Pr. > F	0.0000	0.0173	0.0046	0.0000	0.0000	0.0000	0.0187	0.0000
Root MSE	21.1	29.6	28.2	19.0	17.6	20.3	22.9	16.9

* *p* < 0.001; ** *p* < 0.01; *** *p* < 0.05. †† *p* < .05 after HB correction

Reference categories: 60 to 69 years old; being single (including divorced/separated); very good self-reported financial situation; currently working; no chronic diseases. *PF* physical functioning, *RP* role limitations due to physical health problems, *RE* role limitations due to emotional problems, *VT* vitality, energy and fatigue, *MH* mental health including psychological distress and emotional well-being, *SF* social functioning, *BP* bodily pain, *GH* general health perception

Regression results indicate that higher HRQoL was associated with higher educational attainment, being married/partnered, and having higher social support, while lower HRQoL was associated with being widowed, in worse financial situation, having chronic diseases and being in the oldest age groups. Marital status was statistically significant in the estimated models for five domains, while financial situation was significant in the models for all eight domains of HRQoL. Regarding living arrangements and social support, number of contacts with family members and close friends showed an effect only on physical function (*p* < 0–01) and vitality (*p* < 0–05), but additional household members living with the respondent had no effect on the 8 domains of the SF-36. Finally, with the exception of the general health domain (*p* < 0.01), having chronic diseases had no effect on estimated models on HRQoL domains.

## Discussion

In the past decades, ageing research and knowledge of older persons in Mexico has increased significantly. However, studies on HRQoL and its associated determinants are still scarce, especially regarding studies on minority or ethnic groups within the country. To our knowledge, this is one of the few studies on ethnic minority older adults in Mexico and to address a wide range of determinants of HRQoL in Mexican older adults.

Studying HRQoL is highly relevant when investigating conditions and characteristics of older adults as it helps determine specific health and disability conditions and provides information on the relationship of HRQoL dimensions to different socioeconomic and health factors. In this representative sample of Jewish older adults in Mexico, we found that they present more than double the average number of years of schooling, they report better financial situation, continue to be formally employed in older ages and have higher access to health insurance and private health care services than the average Mexican older adult as reported in the national surveys. All of these factors have been extensively studied as fundamental factors of healthy and successful ageing and their influence in the results of the present study, are also evident.

Jewish older persons in Mexico report values similar to normative data in studies in some high income countries [35–37], with the exception of PF that was lower in our study, and VT that was higher. In addition, compared to other Mexican studies [30, 38] our sample shows higher scores in all dimensions of the SF-36.

In line with previous studies, results from our study show that HRQoL depends on age, educational attainment, marital status, and perception of financial status [17, 39, 40]. In particular in our sample, economic situation measured through self-reported financial situation was significant in the estimated models for all eight domains of HRQoL, while number of contacts with family members and close friends showed an effect only physical function (*p* < 0–01) and vitality (p < 0–05), but additional household members living with the respondent had no effect on the 8 domains of the SF-36. This shows that within social networks, the presence of a spouse and contact with close relatives and friends appear to have a higher positive influence on HRQoL than living arrangements measured as total number of household members. Interestingly, with the exception of the general health domain (*p* < 0.01), having chronic diseases had no effect on any physical or mental health dimensions of HRQoL.

Given the fact that our study sample shows higher scores than other groups of older persons in Mexico and similar scores to those found in higher income countries, some issues are important to consider. First, compared to nationally representative samples, Jewish older persons show much better socioeconomic characteristics. Data from the 2012 wave of the Mexican Health and Aging Study, MHAS [1], for example, show that nationally, adults 60 years and older have much lower educational attainment, than Jewish older persons (13 years’ average), having completed an average of 6 years schooling. Also, they show lower percentages of men and women who still work, and report much worse financial situation that Jewish older persons. Given the observed impact of socioeconomic determinants in HRQoL in previous studies, we can expect this to be one of the main reasons behind these observed differences. As more recent generations of adults in the country show higher educational attainment than their predecessors, and probably better working and income conditions, we could hypothesise that more advantaged conditions as the ones we currently observe in Jewish older adults, may reflect in better HRQoL in future generations of older adults in the country.

Sample respondents also reported being highly socially active either by working, or having frequent contact with family and friends, factors that have been noted as an important determinants of healthy ageing. Based on strong community based organizations, families and individuals are helped in case of need with food, health care, medicine, rent, scholarships, and other issues through a wide array of services and programs, including women’s organizations. It is important in the future to explore if this strong social support has also played a role achieving better health and wellbeing in old age in this community and through which mechanisms. But for now, we could suggest that more attention must be paid to social networks, as well as family and community support given to the elderly, in order to achieve a better HRQoL, as social networks and social support have been shown to have a positive impact on HRQoL [41].

In addition, even when having chronic diseases was not statistically significant in determining scores of several dimensions of HRQoL in this study, in general, Jewish older persons report less prevalence of chronic diseases, with the exception of cancer and hypertension, and much better self-reported health status. Finally, older persons from the Jewish community have much higher percentages of health insurance and use of private health care services which we hypothesize grants them better access to all preventive and curative services, timely diagnosis and better treatment of chronic disease, which in turn is reflected in better overall wellbeing and higher health related quality of life. This could be result of their higher educational attainment which highly likely granted them better jobs within the formal sector thorough out their life-cycle. As better health status could imply better functioning and less impairment caused by chronic disease complications, this can also be the main driver of better self-reported HRQoL in our sample of Jewish older adults, compared to other older adults in the country. In sum, we could hypothesise that by achieving a higher educational level, these individuals had higher access to formal employment with higher income and that in turn allowed them access to better health care services, either through social security institutions or private services, better preventive and curative services and that these, in the end allow them to continue actively working and in better health as reported in their HRQoL scores.

As in most studies, some limitations must be considered when interpreting the results of the study presented here. First, the study’s cross-sectional design limits any causal inference between HRQoL and socio-demographic and health characteristics. Longitudinal studies would allow for this and for estimating long-term effects of different characteristics on HRQoL, however, these are expensive and difficult to perform. Second, while most Jewish older persons still live in the community, this study only included community-dwelling individuals and therefore, results might be biased by leaving out those institutionalised and who may be in worse health. In the future, a study in the Jewish community’s nursing home should be conducted in order to characterize its residents and compare the results to those presented in this study. Finally, the lack of objective performance measures in our study such as weight, height, walking speed or grip strength could be leaving out important covariates in the analysis of additional determinants of HRQoL.

However, the study has also important strengths in being one of the few studies of HRQoL of older persons in Mexico and the first to study a representative sample of older persons of a minority population group usually under- or not represented in national surveys. It extends the use of the SF-36 as a valid tool to measure a fundamental factor of overall wellbeing in older persons, HRQoL, and encourage its wider use in other Mexican regions or with other minority or ethnic groups in the country. In addition, having estimated the scores of the SF-36 for this population group opens the potential to extend our analysis in HRQoL, for example, by deriving the Short Form-6D (SF-6D) classification from the SF-36 which allows, in turn, obtaining quality adjusted life years (QALYs) in a population sample [42]. Notwithstanding the fact that this is a cross-sectional study and inference on causality may not be established, this study presents a clear example of better or successful ageing within the county.

## Conclusions

HRQoL of Jewish older adults in Mexico resembles more that of adults and older adults in higher income countries than that of adults and older adults in other studies in Mexico, presenting a clear example of better or successful ageing within the county. Findings also show that gender, educational attainment, marital status and social support & participation are relevant factors influencing HRQoL. Further studies comparing other characteristics among them could help bring further understanding of these differentiated ageing processes. In addition, results from this study could be used by public health and social sector institutions in the planning of strategies to insure optimal conditions for current adults now and in their ageing process. It will also be important to observe if younger Mexican adults who have achieved better educational attainment and employment opportunities than their parents or grandparents, and similar to what Jewish older persons have now, present social and health characteristics in the future similar to those observed in this study.

## Data Availability

The dataset generated and used for the analyses in the current study are not publicly available due to personal data protection norms as it includes personal identification information. Data could be available from the corresponding author under specific conditions.

## Abbreviations

HRQoL: Health Related Quality of Life
SF-36: Medical Outcomes Study (MOS) 36-item Short Form Health Survey
SF-6D: Short form-6D
QoL: Quality of Life
QALY: Quality-adjusted life year
PF: Physical functioning
RP: Role limitations due to physical health problems
RE: Role limitations due to emotional problems
VT: Vitality, energy and fatigue
MH: Mental health including psychological distress and emotional well-being
SF: Social functioning
BP: Bodily pain
GH: General Health perceptions
MJCC: Mexican Jewish Central Committee
